# Undernutrition and Increased Healthcare Demand: Evidence from a Community-Based Longitudinal Panel Study in Singapore

**DOI:** 10.3390/nu17111781

**Published:** 2025-05-24

**Authors:** Lixia Ge, Chun Wei Yap

**Affiliations:** Health Services and Outcomes Research, National Healthcare Group, Level 4 Annex@NSC, 1 Mandalay Road, Singapore 308205, Singapore; chun_wei_yap@nhg.com.sg

**Keywords:** healthcare utilisation, longitudinal analysis, nutritional assessment, panel data, undernutrition

## Abstract

**Background/Introduction**: Undernutrition’s impact on healthcare utilisation across age groups and care settings remains underexplored, particularly in Asian contexts. This study investigated the dynamic association between nutritional status and healthcare utilisation among community-dwelling adults in Singapore and assessed whether age modified this relationship. **Methods**: The study sampled 1703 adults enrolled in the Population Health Index study. Nutritional status was assessed annually using the Mini Nutritional Assessment, and healthcare utilisation data—across primary care, specialist outpatient clinics (SOCs), emergency departments (EDs), day surgeries, and inpatient admissions—were extracted from administrative databases. Negative binomial regressions with interaction terms using longitudinal panel data were conducted to examine age-modified effects. **Results**: At baseline, 9.7% of participants were classified as undernourished, with a higher prevalence in older adults (15.0%). Key risk factors for undernutrition included female sex, unemployment, financial inadequacy, currently smoking, lack of formal education, and multimorbidity. Undernutrition was associated with increased ED visits (IRR 1.41, AME: 0.35) and inpatient admissions (IRR 1.52, AME: 0.42). Among older adults, undernutrition was associated with less primary and specialist care (IRR: 0.72 and 0.57), while younger undernourished adults had more SOC visits (AME: 0.46). Older undernourished adults had 0.46 more ED visits and 0.47 more inpatient admissions on average in one year, though these increases did not differ from younger adults (interaction *p* > 0.05). **Conclusions**: Undernutrition is associated with increased ED visits and inpatient admissions, especially in older adults. Integrating nutritional screening and targeted interventions into community and primary care may help reduce preventable hospitalisations in high-risk populations.

## 1. Introduction

Despite economic progress and global commitments to improving nutrition, malnutrition (including both overnutrition and undernutrition) or its risk remains as a major public health challenge, particularly among older adults. Furthermore, most countries are off-track in meeting global targets for reducing diet-related non-communicable diseases [1]. Malnutrition and its associated risks negatively affect health and well-being, leading to functional decline, increased morbidity and mortality, and reduced quality of life [2,3,4]. Globally, the prevalence of undernutrition among older adults, as assessed by the Mini Nutritional Assessment (MNA), shows considerable variation from 1.2% [5] to 52.5% [6], with differences attributable to geographic, demographic, and clinical factors, including chronic disease burden [7,8]. A recent systematic review and meta-analysis study estimated that up to 18.6% of older adults worldwide exhibit nutrient deficiencies [8] and community-dwelling elders remain vulnerable to the risk of undernutrition [9]. In Singapore, the rapidly ageing population face a growing but often overlooked risk of malnutrition among community-dwelling adults. Studies reveal that a substantial proportion are at moderate to high risk due to inadequate nutrient intake, poor appetite, chronic conditions, and cognitive impairments [10,11,12].

While malnutrition is frequently examined in older populations, younger adults can be susceptible as well. Emerging evidence suggests that poor diet quality, unhealthy dietary patterns, food insecurity, and lifestyle factors contribute to undernutrition risk across age groups [13,14]. For instance, younger individuals with chronic illnesses, low socioeconomic status, or disordered eating behaviours may develop nutrient deficiencies, increasing their risk of adverse health outcomes [15,16]. However, research and interventions remain disproportionately focused on older adults, who are often perceived as the most vulnerable group. This imbalance has led to gaps in understanding and addressing the risk of undernutrition in younger community-dwelling populations.

A growing body of evidence links undernutrition to adverse health outcomes, including accelerated muscle and bone loss, increased risk of falls and fractures, mobility limitations, and loss of independence, all of which ultimately compromise survival [3,17]. Recent literature has also documented the relationship between undernutrition and elevated healthcare utilisation and costs, particularly among older adults. Poor nutritional status in ageing populations is associated with more utilisation of healthcare resources such as higher use of outpatient services, frequent hospitalisations, and greater emergency care needs, driving up healthcare expenditures [2,3,18].

Despite these advances, critical contextual and methodological gaps remain. First, while undernutrition-related healthcare burdens are well documented in older adults, evidence remains scarce for younger and middle-aged populations—a concerning oversight given rising rates of diet-related chronic diseases (e.g., diabetes, cardiovascular disorders) in these groups, which may exacerbate under recognised nutritional risks. Second, the existing literature disproportionately reflects Western or low-income settings, with limited data from multi-ethnic Asian contexts like Singapore, where dietary habits, healthcare systems, and socioeconomic determinants of nutrition differ substantially. Third, research has typically examined healthcare utilisation in siloed settings (e.g., hospitalisations alone) rather than holistically across primary, specialist, emergency, and inpatient care, potentially obscuring undernutrition’s system-wide burden. Fourth, methodological reliance on prospective designs with single timepoint assessments of nutritional status and healthcare utilisation fails to address two key challenges: (1) pre-existing healthcare utilisation patterns that may confound associations and (2) temporal variability in nutritional status, confounding factors, and care demands. These limitations hinder the identification of optimal intervention windows and obscure the dynamic relationship between nutritional decline and healthcare needs.

This study aims to address these gaps by investigating the dynamic relationship between nutritional status and healthcare utilisation among community-dwelling adults in Singapore, as well as examining how age group modifies these associations. Specifically, the objectives of the study are the following:

- Identifying factors associated with undernutrition among community-dwelling adults in Singapore’s multi-ethnic population.
- Exploring the dynamic association between nutritional status and annual healthcare utilisation using panel data.
- Assessing whether the association between undernutrition and healthcare utilisation differs between young (21–59 years) and older (≥60 years) adults by testing for an interaction between nutritional status and age group.

We hypothesise that undernutrition or poor nutritional status is dynamically associated with elevated healthcare utilisation, manifesting in increased inpatient admissions and emergency department (ED) visits—with effect magnitudes varying by age group and care setting. By leveraging longitudinal data linkage and evaluating multi-setting healthcare endpoints, our findings provide panel data evidence on how undernutrition influences healthcare demands in Singapore’s multi-ethnic adult population, offering actionable insights for targeted screening programmes and resource planning in Singapore’s rapidly ageing society.

## 2. Materials and Methods

### 2.1. Study Design

This longitudinal panel study analysed data from Phase 1 of the Population Health Index (PHI) study, a community-based survey conducted in Singapore’s Central Region with annual data collection over three consecutive years. The PHI study’s survey instruments, stratified random sampling methodology, and survey administration have been previously described [19,20]. In brief, the study population comprised Singapore citizens and permanent residents aged ≥21 years who had resided in randomly selected dwelling units for at least 6 months in the past year. Trained surveyors explained the study, obtained written informed consent from randomly selected household members, and conducted structured face-to-face interviews using standardised and validated questionnaires. Interviews were conducted either during household visits or at alternative locations and times based on participants’ preferences.

From November 2015 to November 2016, a total of 1942 adults (response rate 53.3%) provided written informed consent and completed the baseline assessment (T0). These participants were followed annually, with continued participation remaining voluntary at each stage. Among them, 1703 participants (87.7%) additionally consented to data linkage, enabling their survey responses to be matched with clinical diagnoses and healthcare utilisation records from administrative databases. The PHI study was approved by the ethical committee of the institutional Domain Specific Review Board (Reference Number: 2015/00269).

### 2.2. Study Participants

**Figure 1:**
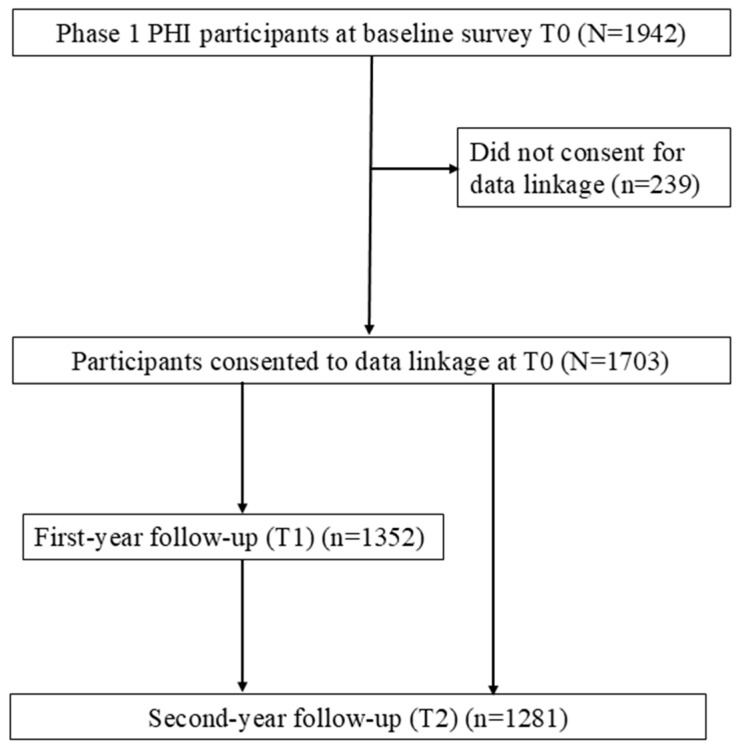
Participant flowchart.

This study sampled 1703 participants who consented to data linkage. Among them, 1352 (79.4%) completed the first-year follow-up (T1), while 1281 (75.2%) completed the second-year follow-up (T2). The remaining participants were lost to follow-up. Most participants (n = 1146, 67.3%) completed all three assessments, 341 (20.0%) completed two assessments, and 216 (12.7%) participated only at baseline. The participant flowchart is illustrated in Figure 1.

### 2.3. Variables and Measurements

#### 2.3.1. Outcomes: Healthcare Utilisation in Different Settings

The annual healthcare utilisation outcomes were measured over the 12-month period following each survey (including nutritional assessment), ensuring temporality between nutritional status and subsequent healthcare utilisation. All utilisation data were obtained from the Population Health Data Mart (PHDM), a centralised data repository developed by Singapore’s National Healthcare Group (NHG), one of the three public health regional systems. The PHDM integrates comprehensive population health data, enabling NHG to create detailed health profiles, segment populations by health indicators, and conduct advanced data analytics for proactive health management.

Annual healthcare utilisation data were extracted for five clinical settings: (1) polyclinics (primary care), (2) specialist outpatient clinics (SOCs), (3) EDs, (4) day surgery (DS), and (5) inpatient admissions. These data were organised relative to survey timepoints into three distinct periods: one-year preceding baseline survey, one-year following each annual survey, and five-year post-baseline.

#### 2.3.2. Exposure: Nutritional Status

The nutritional status of participants at each survey assessment point was determined using the 18-item full Mini Nutritional Assessment (MNA^®^), a validated tool for detecting undernutrition risk in older adults [21]. The MNA^®^ comprises two components: (1) a 6-item screening section (maximum 14 points) and (2) a 12-item assessment section (maximum 16 points). The maximum total nutritional score is 30 points. In this study, undernutrition was defined by a total nutritional score of below 24 out of 30 points (coded as 0). For participants with incomplete assessment data, a screening score of 11 or below (out of 14) was used to identify potential undernourished individuals. The MNA^®^ has demonstrated high reliability and validity in Singapore’s multicultural adult population, with extensive use across clinical and community settings.

#### 2.3.3. Covariates

The study incorporated socio-demographic, lifestyle, and clinical covariates that may confound the relationship between nutritional status and healthcare utilisation. Socio-demographic factors included age, sex (male; female), ethnicity (Chinese; not Chinese), formal educational attainment (yes; no), marital status (single; married; previously married [divorced/separated/widowed]), employment status (employed; unemployed, including retired and inactive), housing type (public 1–2 room; public 3–4 room; public 5+ room/Executive/private), living alone (yes; no), and self-perceived financial adequacy for essential daily living (adequate; inadequate). Lifestyle factors comprised smoking status (never smoked; current smoker; former smoker) and alcohol misuse (yes; no). Clinical characteristics included the number of chronic conditions (0, 1, or ≥2 comorbidities), which were derived based on the number of self-reported diagnosis of 17 chronic conditions [22]. All covariates were collected at each survey, allowing them to be treated as time-varying if changes occurred over time. Sex and ethnicity were considered time-invariant, as they remained constant throughout the study period. In addition, baseline healthcare utilisation across care settings during the 12 months prior to the participant’s first MNA assessment was also extracted.

### 2.4. Statistical Analysis

The extent of missing data at both the timepoint and variable levels was summarised using frequencies and percentages. Missingness due to loss of follow-up increased over time, with 20.6% of participants missing data at T1 and 24.8% at T2. Among participants who completed the surveys, there were no missing values at the variable level.

Descriptive analyses were performed to summarise baseline socio-demographics, lifestyle factors, and multimorbidity for all study participants and for participants with different baseline nutritional statuses. Categorical variables were described using frequencies and percentages, while continuous variables were presented as means and standard deviations (SDs).

Independent *t*-tests were used for normally distributed continuous variables, while Mann–Whitney U tests were applied for non-normally distributed continuous variables. Chi-squared tests were conducted for categorical variables to assess differences in baseline characteristics between the two nutritional groups. Multiple logistic regression was performed to examine the associations between baseline characteristics and the likelihood of undernutrition, adjusting for all other characteristics. Odds ratios (ORs), 95% confidence intervals (CIs), and *p*-values for each characteristic were reported.

To examine the longitudinal association between nutritional status and subsequent annual healthcare utilisation, a random-effects negative binomial regression was performed using complete case panel data, accounting for within-participant correlation across repeated measurements. In each model, the dependent variable was setting-specific one-year healthcare utilisation following each survey assessment, and the independent variable was time-varying nutritional status measured during each survey. All analyses were adjusted for the identified covariates measured in each survey and corresponding baseline healthcare utilisation (Model 1). Additionally, average marginal effects (AMEs) were also derived to estimate the average difference in number of visits. To evaluate age’s potential modification effect on their associations, an interaction term between age group and nutritional status was added to Model 1 (Model 2).

The results were presented as incidence rate ratios (IRRs) with corresponding 95% confidence intervals (CIs) and *p*-values. In addition, AMEs between nutritional status groups with corresponding 95% CIs were also reported. All analyses were conducted using Stata/SE 17.0, with statistical significance set at *p* < 0.05.

The reporting of this study followed the STROBE (Strengthening the Reporting of Observational Studies in Epidemiology) checklist for cohort studies [23] (Table S1).

## 3. Results

### 3.1. Baseline Characteristics of Participants

The mean age of all 1703 participants at baseline was 52.5 ± 17.0 years, ranging from 21 to 97 years old, and 36% were aged 60 years and above. Over half of the participants were females (54.1%). The majority were Chinese (77.9%), were married (61.3%), had received formal education (85.3%), and were employed (62.9%). Most participants lived in public 3- or 4-room flats (64.5%) and did not live alone (88.9%). While 85.1% reported financial adequacy for essential daily living, 14.9% reported financial inadequacy. The majority never smoked (74.0%) and did not report alcohol misuse (74.4%), and above 43% did not report a diagnosis of any of the listed chronic conditions (Table 1).

Among the 1703 participants, 165 (9.7%) were classified as undernourished, with a significantly higher prevalence among older adults (15.0%) compared to younger adults (6.7%, *p* < 0.001). Undernourished participants were generally older (mean age 59.1 vs. 51.8 years, *p* < 0.001), with a higher proportion of females (64.9% vs. 52.9%, *p* = 0.003), and had lower educational attainment (35.2% vs. 12.6% with no formal education, *p* < 0.001). They also exhibited higher rates of unemployment (67.3% vs. 33.9%), multimorbidity (61.8% vs. 34.7%), and inadequate financial resources (30.3% vs. 13.2%) compared to their well-nourished peers (*p* < 0.001 for all). Baseline healthcare utilisation (one-year preceding the baseline survey) was consistently higher among undernourished individuals across all settings (*p* ≤ 0.039), except for day surgery visits (*p* = 0.059).

### 3.2. Baseline Factors Associated with Undernutrition

The multiple logistic regression analysis results in Table 2 reveal that female sex (OR 2.20, 95% CI 1.40–3.44), being unemployed/retired/inactive (OR 2.91, 95% CI 1.94–4.38), financial inadequacy (OR 1.94, 95% CI 1.28–2.95), and current smoking (OR 2.70, 95% CI 1.56–4.67) were strongly associated with a higher risk of undernutrition. In contrast, formal education (OR 0.54, 95% CI 0.34–0.85) and being married (OR 0.41, 95% CI 0.26–0.67) were associated with a lower risk of undernutrition. The presence of ≥2 chronic conditions also had a positive association with increased undernutrition risk (OR 1.91, 95% CI 1.17–3.14). Age and ethnicity were not statistically significant factors (*p* > 0.05).

### 3.3. Longitudinal Association Between Nutritional Status and Healthcare Utilisation Using Panel Data

Undernutrition was dynamically associated with increased ED visits (IRR 1.41, 95% CI 1.09–1.84, *p* = 0.010) and inpatient admission (IRR 1.52, 95% CI 1.11–2.10, *p* = 0.011) after adjusting for baseline covariates and corresponding baseline utilisation (Table 3). In absolute terms, undernourished individuals experienced an average of 0.35 additional ED visits and 0.42 more inpatient admissions compared to their well-nourished counterparts. In contrast, no significant associations were observed for primary care visits or day surgery procedures (all *p* > 0.05).

### 3.4. Interaction Effect of Age and Nutritional Status on Healthcare Utilisation

The interaction between age group and nutritional status (Table 4, Model 2) revealed that after adjusting for covariates and baseline healthcare utilisation, the effect of undernutrition on primary care visits at polyclinics and SOC visits differed in the two age groups: undernutrition was associated with fewer primary care (IRR: 0.72, 95% CI: 0.52–0.99, *p* = 0.042) and SOC visits (IRR: 0.57, 95% CI: 0.40–0.80, *p* = 0.001) in older adults but more SOC visits in younger adults (AME: 0.46, *p* = 0.005). Older undernourished adults also reported more ED visits (AME: 0.49, *p* = 0.001) and inpatient admissions (AME: 0.47, *p* = 0.008), though these effects did not statistically differ from younger adults (interaction *p* > 0.05). Undernutrition was not associated with day surgery utilisation in either age groups.

## 4. Discussion

This longitudinal panel study delineates the prevalence and associated factors of undernutrition among Singapore’s community-dwelling adults and provides robust evidence on its temporal and dynamic relationships with healthcare utilisation, including age-specific variations. Our findings show that undernutrition disproportionately affects older adults (15.0% vs. 6.7% in younger adults) and drives distinct utilisation patterns: older undernourished adults exhibited fewer SOC visits but more ED and inpatient care utilisation compared to their younger, well-nourished counterparts. By employing panel data analysis across multiple healthcare settings, this study addresses critical evidence gaps regarding undernutrition’s healthcare burden in Asian populations, its longitudinal progression, and the modifying role of age.

Using mainly the full MNA, our study identified a 15.0% prevalence of undernutrition among older adults (≥60 year), which is comparable to that reported in a Spanish community-based longitudinal study [2] and exceeds the 3.9% reported in an Australian study [24] but is lower than the 23.5% derived from Japanese data [25]—all determined using the MNA six-item screening tool. These variations likely reflect both methodological differences (full vs. short-form assessment) and population characteristics, with our community-dwelling sample potentially capturing less severe but concerning nutritional risk. While older adults remain the most vulnerable group for undernutrition, younger individuals with comorbidities or socioeconomic disadvantages also face significant undernutrition risks, echoing emerging evidence of undernutrition’s lifecycle persistence. Singapore’s distinct context—combining rapid ageing, dietary diversity, and a high level of healthcare access—may explain the intermediate prevalence, underscoring the need for population-specific nutritional screening strategies.

Consistent with the global literature [26,27,28,29], female sex, lack of formal education, unemployment, perceived financial inadequacy, and multimorbidity were found to be associated with undernutrition in the study population. The 2.9-fold higher odds among unemployed/retired/inactive individuals mirrors findings from another local study [10], highlighting the role of economic stability in nutritional access. Notably, smoking tripled the risk of undernutrition (OR 2.70), a finding corroborated by studies linking tobacco use to appetite suppression and nutrient malabsorption [30]. Conversely, marriage and formal education were protective towards undernutrition, likely via conferring social support and better health literacy [31]. Our results extend prior work by identifying these factors in a multi-ethnic developed Asian population, where cultural norms (e.g., familial caregiving) may modulate their effects.

Consistent with findings from European studies [2,18], undernutrition was longitudinally associated with increased ED visits and inpatient admissions, but with comparatively less—though statistically non-significant—utilisation of polyclinic (primary care) clinics, SOCs, and DS centres over time, even after adjusting for covariates and corresponding baseline healthcare utilisation. These patterns may reflect delayed care-seeking among undernourished individuals. Importantly, these results highlight undernutrition’s impact on healthcare demand, underscoring its role as a modifiable risk factor for preventable and high-cost ED visits and hospitalisations—an effect particularly pronounced among undernourished older adults.

These findings align with global evidence suggesting that undernourished older adults living in the community face systemic barriers to accessing primary and specialist care due to multiple reasons (e.g., mobility limitations, under-recognition of undernutrition symptoms, or transport challenges), leading to delayed care-seeking until acute crises necessitate emergency care or hospitalisations [3]. Notably, the observed reduction in primary and SOC visits among older undernourished adults contrasted with the increased SOC utilisation in younger undernourished adults (+0.46 visits). This paradox may reflect distinct care-seeking patterns: younger adults might proactively seek specialist care for early symptoms, while older adults delay care until conditions escalate.

Importantly, physical frailty may be a key pathway linking undernutrition to increased healthcare utilisation. Frailty, which often co-exists with undernutrition, could function both as a mediator and a moderator in this relationship. As a mediator, frailty may develop as a consequence of chronic nutritional deficits, increasing vulnerability to acute health events and hospitalisation. As a moderator, frailty may amplify the impact of undernutrition by compounding physiological and functional decline, thereby amplifying the need for care. Prior studies have demonstrated strong associations between frailty and adverse outcomes such as inpatient admissions and ED visits, suggesting that undernutrition and frailty may interact to escalate healthcare demands [32,33,34]. Future studies should explore these mechanisms using formal mediation/moderation analyses and incorporate direct frailty assessments to refine risk stratification.

The lack of age interaction for inpatient admissions (*p* = 0.440) suggests that undernutrition elevates hospitalisation risk similarly across age groups. However, the absolute effect was greater in older adults (+0.47 admissions, *p* = 0.008), likely due to their greater baseline vulnerability. This underscores undernutrition as a modifiable risk factor for preventable hospitalisations, particularly in ageing populations.

To the best of our knowledge, this is the first study investigating the dynamic association between undernutrition and healthcare utilisation across different care settings among community-dwelling adults in Singapore. The strengths of the study include its longitudinal panel design, multi-setting prospective utilisation data over five years, and robust adjustment for covariates, including baseline setting-specific utilisation. Nevertheless, several limitations should be acknowledged. Residual confounding (e.g., unmeasured factors like social support) may influence the observed associations. Additionally, reliance on self-reported MNA scores may introduce reporting bias. The consent-based data linkage, while achieving a relatively high participation rate (87.7%), could still introduce selection bias, although the demographic comparability of participants to national profiles helps to mitigate this concern. Furthermore, the utilisation data captured in this study were limited to NHG institutions and did not include healthcare usage in the other two regional health systems or private providers, potentially underestimating the total burden of undernutrition. Finally, generalisability to other Asian populations warrants further verification, and the observational nature of the study precludes causal inference.

## 5. Implications for Public Heath Practice

Our findings underscore the critical impact of undernutrition on healthcare utilisation patterns and offer several actionable directions for public health practice in Singapore. First, the integration of routine nutritional screening into primary care and community settings is essential, particularly for high-risk subgroups such as women, individuals without formal education, those who are unemployed, retired, or economically inactive, current smokers, and individuals with multiple chronic conditions. Early identification of undernutrition in these vulnerable populations may enable timely intervention and reduce downstream burden on healthcare services.

Second, tailored intervention strategies should be implemented to address specific access barriers. For instance, older adults facing mobility limitations or transportation challenges could benefit from mobile health clinics or home-based nutritional services that deliver personalised support while improving reach and adherence. Financially vulnerable individuals who struggle to access healthy food due to limited income or social support could benefit from targeted food assistance and community nutrition programmes, such as Share-A-Pot, which offer both nutritional and social support.

These recommendations align with Singapore’s population health management strategy, which emphasises preventive care and integrated chronic disease management within the community. By embedding nutritional care as a foundational component of these efforts, public health systems can better address the modifiable risk of undernutrition and reduce avoidable hospital admissions.

## 6. Conclusions

Undernutrition was associated with increased ED visits and inpatient admissions among community-dwelling adults. The disparity in the association between undernutrition and healthcare utilisation across care settings between young and older adults highlights potential gaps in early care access and underscores undernutrition as a critical, yet modifiable, risk factor for avoidable hospitalisations. The findings support the integration of routine nutritional screening and targeted interventions into primary care and community settings, particularly for high-risk groups, to enable timely detection and management.

## Figures and Tables

**Table 1:** Baseline characteristics of study participants, overall and by nutritional status.

Characteristics	Total (n = 1703)	Normal (n = 1538)	Undernourished (n = 165)	*p*-Value
**Age in years, mean ± SD**	52.5 ± 17.0	51.8 ± 16.3	59.1 ± 21.0	<0.001
**Age group**				<0.001
21–59, n (%)	1090 (64.0)	1017 (66.1)	73 (44.2)	
≥60, n (%)	613 (36.0)	521 (33.9)	92 (55.8)	
**Sex**				0.003
Male, n (%)	782 (45.9)	724 (47.1)	58 (35.2)	
Female, n (%)	921 (54.1)	814 (52.9)	107 (64.9)	
**Ethnicity**				0.618
Chinese, n (%)	1326 (77.9)	1195 (77.7)	131 (79.4)	
Not Chinese, n (%)	377 (22.1)	343 (22.3)	34 (20.6)	
**Marital status**				<0.001
Single, n (%)	387 (22.7)	343 (22.3)	44 (26.7)	
Married, n (%)	1044 (61.3)	966 (62.8)	78 (47.3)	
Previously married, n (%)	272 (16.0)	229 (14.9)	43 (26.1)	
**Formal educational attainment**				<0.001
No, n (%)	251 (14.7)	193 (12.6)	58 (35.2)	
Yes, n (%)	1452 (85.3)	1345 (87.5)	107 (64.9)	
**Employment status**				<0.001
Employed, n (%)	1071 (62.9)	1017 (66.1)	54 (32.7)	
Unemployed, n (%)	632 (37.1)	521 (33.9)	111 (67.3)	
**Housing type**				<0.001
Public 1/2 room, n (%)	166 (9.8)	136 (8.8)	30 (18.2)	
Public 3/4 room, n (%)	1098 (64.5)	995 (64.7)	103 (62.4)	
Public 5+ room/Executive/private, n (%)	439 (25.8)	407 (26.5)	32 (19.4)	
**Living alone**				0.935
No, n (%)	1514 (88.9)	1367 (88.9)	147 (89.1)	
Yes, n (%)	189 (11.1)	171 (11.1)	18 (10.9)	
**Perceived financial adequacy**				<0.001
Adequate, n (%)	1450 (85.1)	1335 (86.8)	115 (69.7)	
Inadequate, n (%)	253 (14.9)	203 (13.2)	50 (30.3)	
**Smoking status**				0.014
Never smoked, n (%)	1260 (74.0)	1151 (74.8)	109 (66.1)	
Current smoker, n (%)	230 (13.5)	196 (12.7)	34 (20.6)	
Former smoker, n (%)	213 (12.5)	191 (12.4)	22 (13.3)	
**Alcohol misuse**				0.122
No, n (%)	1267 (74.4)	1136 (73.9)	131 (79.4)	
Yes, n (%)	436 (25.6)	402 (26.1)	34 (20.6)	
**Number of chronic conditions**				<0.001
0, n (%)	739 (43.4)	695 (45.2)	44 (26.7)	
1, n (%)	329 (19.3)	310 (20.2)	19 (11.5)	
≥2, n (%)	635 (37.3)	533 (34.7)	102 (61.8)	
**Baseline healthcare utilisation**, mean ± SD; median (Q1–Q3)				
Polyclinic visits	1.4 ± 2.9; 0 (0-1)	1.3 ± 2.7; 0 (0-1)	2.0 ± 4.5; 0 (0-3)	0.039
SOC visits	1.4 ± 4.1; 0 (0-0)	1.2 ± 3.6; 0 (0-0)	3.3 ± 6.9; 0 (0-4)	<0.001
ED visits	0.2 ± 0.6; 0 (0-0)	0.1 ± 0.5; 0 (0-0)	0.6 ± 1.3; 0 (0-1)	<0.001
DS procedures	0.1 ± 0.4; 0 (0-0)	0.1 ± 0.4; 0 (0-0)	0.1 ± 0.4; 0 (0-0)	0.059
Inpatient admissions	0.1 ± 0.4; 0 (0-0)	0.1 ± 0.3; 0 (0-0)	0.3 ± 0.9; 0 (0-0)	<0.001

The percentages are reflected as column percentages. Abbreviations: DS—day surgery; ED—emergency department; Q1—25th percentile; Q3—75th percentile; SOC—specialist outpatient clinic.

**Table 2:** Factors associated with undernutrition.

Baseline Characteristics	Odds Ratio	95% Confidence Interval	*p*-Value
**Age in years**	1.00	0.99, 1.02	0.950
**Female**	2.20	1.40, 3.44	0.001
**Chinese (reference: not Chinese)**	1.25	0.79, 1.98	0.347
**Formal educational attainment (reference: no)**	0.54	0.34, 0.85	0.008
**Marital status (reference: single)**			
Married	0.41	0.26, 0.67	<0.001
Previously married	0.56	0.31, 1.02	0.058
**Unemployed** **(reference: employed)**	2.91	1.94, 4.38	<0.001
**Housing type (reference: public 1/2 rooms)**			
Public 3/4 rooms	0.72	0.43, 1.23	0.236
Public 5+ rooms/executive/private	0.66	0.35, 1.25	0.201
**Living alone (reference: no)**	0.46	0.25, 0.84	0.012
**Perceived financial inadequacy (reference: adequate)**	1.94	1.28, 2.95	0.002
**Smoking status (reference: never smoked)**			
Current smoker	2.70	1.56, 4.67	<0.001
Former smoker	1.63	0.91, 2.91	0.102
**Alcohol misuse (reference: no)**	1.07	0.68, 1.70	0.761
**Number of chronic conditions (reference: 0)**			
1	0.84	0.47, 1.51	0.553
≥2	1.91	1.17, 3.14	0.010

**Table 3:** Longitudinal association between nutritional status and healthcare utilisation using panel negative binomial regression analysis (Model 1).

One-Year Healthcare Utilisation	IRR (95% CI)	AME (95% CI)	*p*-Value
Polyclinic visits	0.93 (0.81, 1.07)	−0.07 (−0.21, 0.07)	0.339
SOC visits	0.98 (0.85, 1.13)	−0.02 (−0.16, 0.12)	0.776
ED visits	1.41 (1.09, 1.84)	0.35 (0.08, 0.61)	0.010
DS procedures	0.64 (0.36, 1.15)	−0.44 (−1.02, 0.14)	0.137
Inpatient admissions	1.52 (1.10, 2.10)	0.42 (0.10, 0.74)	0.011

Adjusted for age group, timepoint, female sex, Chinese nationality, marital status, formal education, living alone, perceived financial adequacy, smoking status, alcohol misuse, number of chronic conditions, and corresponding baseline healthcare utilisation. Abbreviations: AME—average marginal effect; CI—confidence interval; DS—day surgery; ED—emergency department; IRR—incidence rate ratio; SOC—specialist outpatient clinic.

**Table 4:** Age and nutritional status interaction effects on healthcare utilisation (Model 2).

One-Year Healthcare Utilisation	Interaction Between Age and Nutritional Status		Undernourished Younger Adults (Aged 21–59)		Undernourished Older Adults (Aged ≥ 60)	
	IRR (95% CI)	*p*-Value	AME (95% CI)	*p*-Value	AME (95% CI)	*p*-Value
Polyclinic visits	0.72 (0.52, 0.99)	0.042	0.18 (−0.09, 0.46)	0.195	−0.15 (−0.32, 0.01)	0.070
SOC visits	0.57 (0.40, 0.80)	0.001	0.46 (0.14, 0.77)	0.005	−0.11 (−0.26, 0.04)	0.158
ED visits	1.73 (0.96, 3.09)	0.067	−0.05 (−0.57, 0.46)	0.837	0.49 (0.19, 0.79)	0.001
DS procedures	0.80 (0.22, 2.83)	0.725	−0.27 (−1.36, 0.81)	0.622	−0.50 (−1.18, 0.18)	0.147
Inpatient admissions	1.36 (0.62, 3.00)	0.440	0.16 (−0.57, 0.89)	0.661	0.47 (0.12, 0.83)	0.008

Model 2: independent variable is interaction term between age group and nutritional status, adjusted for timepoint, female sex, Chinese nationality, marital status, formal education, living alone, perceived financial adequacy, smoking status, alcohol misuse, number of chronic conditions, and corresponding baseline healthcare utilisation. Abbreviations: AME—average marginal effect, CI—confidence interval, DS—day surgery, ED—emergency department, IRR—incidence rate ratio; SOC—specialist outpatient clinic.

## Data Availability

According to the Data Protection Act Commission Singapore—Advisory Guidelines for the Healthcare Sector, the personal health data collected for the Population Health Index study are not publicly available due to legal and ethical restrictions related to data privacy protection. However, the minimal dataset underlying the findings in the manuscript are available upon request to interested researchers after authorization by the institutional ethical committee. Interested researchers may contact Dr Chun Wei Yap (chun_wei_yap@nhg.com.sg) for data requests.

## Supplementary Materials

The following supporting information can be downloaded at: https://www.mdpi.com/article/10.3390/nu17111781/s1, Table S1. STROBE Statement—Checklist of items that should be included in reports of cohort studies.

## Abbreviations

The following abbreviations are used in this manuscript:

AME	Average marginal effect
CI	Confidence interval
DS	Day surgery
ED	Emergency department
IRR	Incidence rate ratio
MNA	Mini Nutritional Assessment
NHG	National Healthcare Group
OR	Odds ratio
PHDM	Population Health Data Mart
Q1	25th percentile
Q3	75th percentile
SD	Standard deviation
SOC	Specialist outpatient clinic
